# Natural Frequencies Optimization of Thin-Walled Circular Cylindrical Shells Using Axially Functionally Graded Materials

**DOI:** 10.3390/ma15030698

**Published:** 2022-01-18

**Authors:** Nabeel Taiseer Alshabatat

**Affiliations:** Mechanical Engineering Department, Tafila Technical University, Tafila 66110, Jordan; nabeel@ttu.edu.jo

**Keywords:** cylindrical shell, functionally graded material, natural frequency, optimization

## Abstract

One method to avoid vibration resonance is shifting natural frequencies far away from excitation frequencies. This study investigates optimizing the natural frequencies of circular cylindrical shells using axially functionally graded materials. The constituents of functionally graded materials (FGMs) vary continuously in the longitudinal direction based on a trigonometric law or using interpolation of volume fractions at control points. The spatial change of material properties alters structural stiffness and mass, which then affects the structure’s natural frequencies. The local material properties at any place in the structure are obtained using Voigt model. First-order shear deformation theory and finite element method are used for estimating natural frequencies, and a genetic algorithm is used for optimizing material volume fractions. To demonstrate the proposed method, two optimization problems are presented. The goal of the first one is to maximize the fundamental frequency of an FGM cylindrical shell by optimizing the material volume fractions. In the second problem, we attempt to find the optimal material distribution that maximizes the distance between two adjoining natural frequencies. The optimization examples show that building cylindrical shells using axially FGM is a useful technique for optimizing their natural frequencies.

## 1. Introduction

Cylindrical shells are extensively employed in various applications such as aerospace, automobile, marine, and construction industries. They are usually exposed to dynamic excitations, which cause vibrations and make noise. Reducing vibrations and noise radiation from these structures are important issues in the early stages of structural design process. A common technique to decrease vibrations is shifting the natural frequencies of a structure away from the excitation frequencies to prevent resonance. Different passive methods are employed for optimizing the natural frequencies of cylindrical shells, and these include adjusting the thickness, adding stiffeners, and tailoring material. For example, Alzahabi [1] shifted the natural frequencies of a submarine hull by controlling the local thickness. The length of the hull was divided into equal segments, and then the thickness of each segment was optimized to achieve the sought natural frequency. Nasrekani et al. [2] studied the effect of changing the thickness, continuously along a cylindrical shell, on its first axisymmetric natural frequency. Bagheri et al. [3] investigated a multi-objective problem for simultaneously maximizing the fundamental frequency and minimizing the weight of cylindrical shell using ring stiffeners. Mehrabami et al. [4] maximized the fundamental frequency-to-weight ratio of cylindrical shell using rings and strings. Akl et al. [5] minimized the vibration and/or the sound radiation from under water cylindrical shells using stiffeners. In addition to optimizing the natural frequencies, the literature about the optimization of circular cylindrical shells with other objective functions is very wide. For example, Biglar et al. [6] optimized the locations and orientations of piezoelectric patches on cylindrical shells to reduce the vibration. Sadeghifar et al. [7] studied maximizing the critical buckling load and minimizing the weight of orthogonally stiffened cylindrical shells. Belradi et al. [8] utilized anisogrid composite lattice structures in building circular cylindrical shells to optimize the critical buckling load with strength and stiffness constraints.

Laminated composite materials are used in building circular cylindrical shells due to their high stiffness-to-weight ratio. Various studies have dealt with the vibrations of laminated composite cylindrical shells [9,10,11,12,13,14,15,16,17]. The optimization of the stacking sequence for natural frequency optimization is a common technique. For example, Hu and Tsi [18] investigated the maximization of the fundamental frequency for cylindrical shells with and without cutout. The optimization problem was solved using Golden section method. Koide and Luersen [19] used ant colony optimization to maximize the fundamental frequency. Trias et al. [20] investigated the maximization of the fundamental frequency using bound formulation. Miller and Ziemiański [21,22] maximized the fundamental frequency and the distance between two adjoining natural frequencies using the genetic algorithm (GA) in combination with neural networks. Recently, Jing [23] used sequential permutation search algorithm to maximize the fundamental frequency. The fundamental frequency of laminated composite cylindrical shells reinforced with shape memory alloy fibers was investigated by Nekouei et al. [24]. They showed that a small amount of shape memory alloy fibers increased the fundamental frequency significantly.

Due to sudden change in the mechanical properties between laminars, laminated composites are very prone to interlamination damages. Thus, using functionally graded materials (FGMs) overcomes the disadvantages of using multilayer composites, such as delamination and stress concentration [25]. FGMs are composites typically built from two materials with continuous variation of the materials’ composition through the dimensions of structures. These continuous variations of compositions results in a smooth variation of material properties. Owing to their distinct characteristics, FGMs are used in the design optimization of structures [26,27,28]. Several researchers investigated the free vibration of thin-walled FGMs cylindrical shells. Loy et al. [29] investigated the free vibration of simply-supported FGM cylindrical shell utilizing Love’s shell theory and Rayleigh–Ritz method. Using a similar approach, Arshad et al. [30,31] studied FGM cylinders with various boundary conditions. Using first-order shear deformation theory (FSDT) and Rayleigh–Ritz method, Sue et al. [32] investigated a unified solution technique to estimate the natural frequencies of cylindrical and conical shells. Jin et al. [33] employed FSDT and Haar wavelet discretization. Punera and Kant [34] used several higher-order theories and Navier’s method to investigate the free vibration of open FGM cylinders. Ni et al. [35] adopted Reissner shell theory and the Hamiltonian method to find the natural frequencies of FGM cylinders set in elastic mediums. Liu et al. [36] used FSDT and wave function expansions to find the natural frequencies. Alshabatat and Zannon [37] studied the free vibrations of FGM cylindrical shell by employing third-order shear deformation theory and Carrera’s unified formulation. All previous studies on materials’ volume fraction variations in the radial direction of cylindrical shells (i.e., through thickness) based on simple power law [29,30,31,34,35,37], exponential law [30,31], trigonometric law [30,31], and four-parameter power laws [32,33,36].

To the best of our knowledge, the optimization of natural frequencies of thin-walled cylindrical shells using axial grading material has not been attempted yet. Hence, in this work, analyses and optimization of FGM cylindrical shells are presented. First, the effect of material gradient through the axial direction on the natural frequencies of thin cylindrical shells is investigated. Then, the optimization of the natural frequencies is conducted. The material volume fractions are graded through the length of the cylinders according to control points method and a trigonometric law. FSDT and finite element method (FEM) are employed to find the natural frequencies. To validate the present method, numerical results are compared with those available in the literature. A genetic algorithm (GA) is utilized to look for the ideal material distributions that optimize the natural frequencies. Two optimization problems are considered to show the efficiency of the presented method. The methods for the manufacturing and processing of FGMs are not considered here and can be found in [38,39,40].

## 2. Materials and Methods

### 2.1. Functionally Graded Material

Consider a circular cylindrical shell made from FGM, which consists of two materials mixture. The constituents’ volume fractions are graded in the axial direction of the cylinder and follow a simple power law distribution or a trigonometric distribution. The volume fraction of the first material is given by

(1) $$V_{1}(x)={\left(\frac{x}{L}\right)}^{\gamma }$$

(2) $$\mathrm{or}\;\;V_{1}(x)={\left(\alpha +\alpha \cos\left(\eta x+\phi \right)\right)}^{\gamma }$$

where *L* is the length of the cylinder, and *x* is the axial coordinate (0 ≤ *x* ≤ *L*). γ, α, η, and ϕ are the design variables used to control the volume fraction profile. These parameters are chosen such that 0 ≤ *V*_1_(*x*) ≤ 1. Note that $V_{2}\left(x\right)=1-V_{1}\left(x\right)$. In addition to these laws, this work suggests using control points method for optimization problems. In this method, the volume fraction profile is controlled by the volume fractions at some control points that are distributed evenly through the length of the cylinder. The location of *i*th control point, along the cylinder length, is $x_{i}=\left(i-1\right)L/\left(N-1\right)$ in which N is the number of control points. The volume fraction distribution in the cylinder, $V_{1}(x)$, can then be estimated by the piecewise cubic hermit interpolating polynomial (PCHIP) [41,42]. The design variables in this case are $V_{1,1}$, $V_{1,2}$, …, $V_{1,N}$.

The local material properties of FGM are estimated using Voigt model (or the rule of mixture) as

(3) $$E(x)=(E_{1}-E_{2})V_{1}(x)+E_{2},$$

(4) $$\rho (x)=(\rho_{1}-\rho_{2})V_{1}(x)+\rho_{2},$$

(5) $$\nu (x)=(\nu_{1}-\nu_{2})V_{1}(x)+\nu_{2}$$

where *E*_*i*_ is the Young’s modulus, $\rho_{i}$ is the density, $\nu_{i}$ is the Poisson’s ratio, and the subscript i denotes material 1 or 2.

### 2.2. Kinematic Relations

**Figure 1:**
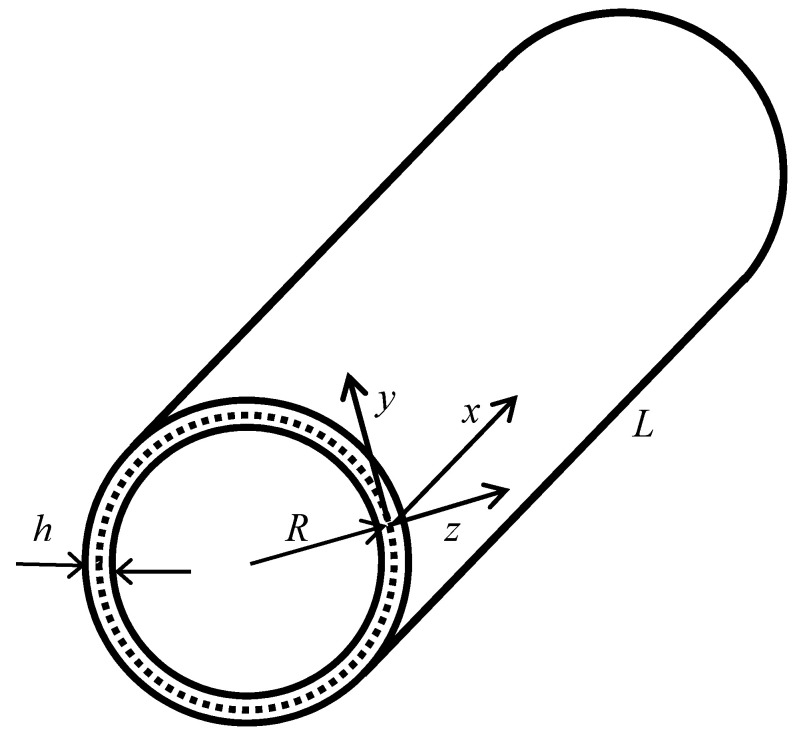
The geometry of the circular cylindrical shell.

A circular cylindrical FGM shell is considered with the length *L*, thickness h, and radius R as shown in Figure 1. The shell is assumed thin (i.e., h/R ≤0.05). According to Reddy et al. [43,44], the solution of Love–Kirchhoff theory is not accurate for composite structures. Thus, FSDT is used in this study. Based on FSDT, the displacements of any point in the cylinder can be written as

(6) $$u\left(x,y,z,t\right)≃u_{o}\left(x,y,t\right)+z\theta_{x}\left(x,y,t\right)$$

(7) $$v\left(x,y,z,t\right)≃v_{o}\left(x,y,t\right)+z\theta_{y}\left(x,y,t\right)$$

(8) $$w\left(x,y,z,t\right)≃w_{o}\left(x,y,t\right)$$

where u, v, and w are the displacements in the axial (x), circumferential (y), and radial (z) directions, respectively. $u_{o}$, $v_{o}$, and $w_{o}$ are the middle surface displacements. $\theta_{x}$ and $\theta_{y}$ are the middle surface rotations, and t is the time.

The strain–displacement relations for thin shells are given as [45]

(9) $$\left\{\varepsilon \right\}=\left\{\begin{array}{c} \varepsilon_{xx}\\ \varepsilon_{yy}\\ \gamma_{xy}\\ \gamma_{xz}\\ \gamma_{yz} \end{array}\right\}=\left\{\begin{array}{c} \frac{\partial u_{o}}{\partial x}+z\frac{\partial \theta_{x}}{\partial x}\\ \frac{\partial v_{o}}{\partial y}+z\frac{\partial \theta_{y}}{\partial y}+\frac{w_{o}}{R}\\ \frac{\partial v_{o}}{\partial x}+\frac{\partial u_{o}}{\partial y}+z\left(\frac{\partial \theta_{y}}{\partial x}+\frac{\partial \theta_{x}}{\partial y}\right)\\ \frac{\partial w_{o}}{\partial x}+\theta_{x}\\ \frac{\partial w_{o}}{\partial y}-\frac{v_{o}}{R}+\theta_{y} \end{array}\right\}.$$

The stress $\sigma_{zz}$ is negligible for a thin shell. The stress–strain relations of FGM cylindrical shell are given as

(10) $$\left\{\begin{array}{c} \sigma_{xx}\\ \sigma_{yy}\\ \tau_{xy}\\ \tau_{xz}\\ \tau_{yz} \end{array}\right\}=\frac{E(x)}{1-\nu^{2}(x)}\left[\begin{array}{ccccc} 1 & \nu (x) & 0 & 0 & 0\\ \nu (x) & 1 & 0 & 0 & 0\\ 0 & 0 & \frac{1-\nu (x)}{2} & 0 & 0\\ 0 & 0 & 0 & \frac{1-\nu (x)}{2} & 0\\ 0 & 0 & 0 & 0 & \frac{1-\nu (x)}{2} \end{array}\right]\left\{\begin{array}{c} \varepsilon_{xx}\\ \varepsilon_{yy}\\ \gamma_{xy}\\ \gamma_{xz}\\ \gamma_{yz} \end{array}\right\}$$

(11) or      {σ}=[Q]{ε}.

The elements of matrix Q are functions of the longitudinal coordinate (x). The governing equations are obtained by applying Hamilton’s principle as

(12) $$\int_{t_{1}}^{t_{2}}\delta \left(T-U\right)dt=0$$

where T and U are the kinetic and strain energies of the vibrating cylindrical shell, respectively. The kinetic energy can be given by

(13) $$T=\frac{1}{2}∭\rho (x)\left[{\left(\frac{\partial u}{\partial t}\right)}^{2}+{\left(\frac{\partial v}{\partial t}\right)}^{2}+{\left(\frac{\partial w}{\partial t}\right)}^{2}\right]dV,$$

and the strain energy can be given by

(14) $$U=\frac{1}{2}∭\sigma \varepsilon dV.$$

The equations of motion are acquired by substituting Equations (13) and (14) into Equation (12). Owing to the complication of the present case, FEM is adopted to solve the equations and find the natural frequencies. A quadrilateral shell element with four nodes is employed to discretize the FGM cylinders. Each node has five degrees of freedom. The material properties of each finite element are apportioned by Equations (3)–(5), in which $V_{1}(x)$ represents the volume fraction of the first material at the center of the element. The degrees of freedom include three translational ($u_{o}^{i}$, $v_{o}^{i}$, and $w_{o}^{i}$) and two rotational ($\theta_{x}^{i}$ and $\theta_{y}^{i}$). The element kinetic and strain energies are given by [46]

(15) $$T_{e}=\frac{1}{2}{{\left\{\dot{\delta }\right\}}_{e}}^{T}{\left[m\right]}_{e}{\left\{\dot{\delta }\right\}}_{e}$$

(16) $$\;\;\;\mathrm{and}\;\;\;\;U_{e}=\frac{1}{2}{\left\{\delta \right\}}_{e}{}^{T}{\left[k\right]}_{e}{\left\{\delta \right\}}_{e},$$

where ${\left\{\delta \right\}}_{e}{}^{T}=\left\{\delta_{1}^{T},\delta_{2}^{T},\delta_{3}^{T},\delta_{4}^{T}\right\}$, $\delta_{i}^{T}=\left\{u_{o}^{i},v_{o}^{i},w_{o}^{i},\theta_{x}^{i},\theta_{y}^{i}\right\}$, ${\left[m\right]}_{e}$ is the element mass matrix, and ${\left[k\right]}_{e}$ is the element stiffness matrix. The element mass matrix is given by

(17) $${\left[m\right]}_{e}=∭\rho N^{T}N\left|J\right|dV_{e},$$

and the element stiffness matrix is

(18) $${\left[k\right]}_{e}=∭B^{T}\bar{Q}B\left|J\right|dV_{e},$$

where N is the shape functions matrix, J is the Jacobian matrix, B is the strain-displacement matrix, and $\bar{Q}$ is the stress-strain matrix with respect to global coordinates. The shape functions are quadratic. Equations (17) and (18) can be evaluated using Gause–Legendre numerical integration. Equation (17) can be evaluated using 3 × 3 integration points in the in-plane coordinates [46]. For thin shells, Equation (18) can be evaluated using 2 × 2 integration points in the in-plane coordinates [47]. The details of the finite element formulations are given in [46]. The governing equations for the free vibration of FGM cylinders are given as

(19) $$\left[M\right]\left\{\ddot{\delta }\right\}+\left[K\right]\left\{\delta \right\}=\left\{0\right\},$$

where [M] and [K] are the global mass and stiffness matrices, respectively, and {δ} and $\left\{\ddot{\delta }\right\}$ are the nodal displacement and acceleration vectors, respectively. The natural frequencies ($\omega_{n}$) and mode shapes can be found by solving the following eigenvalue problem:

(20) $$\left[K-\omega_{n}{}^{2}M\right]\left\{\delta \right\}=\left\{0\right\}.$$

Note that the fundamental frequency is the smallest value of $\omega_{n}$.

## 3. Results and Discussion

### 3.1. Validation and Convergence

In this study, FEM is employed for free vibration analysis of cylindrical shells. The eigenvalue problem (Equation (20)) is solved using Block Lanczos iteration method [48], which is performed using in-house program developed with MATLAB software. To evaluate the accuracy of the algorithm in the present method, validation and convergence study is performed for clamped-free (CF) isotropic circular cylindrical shells with Naeem and Sharma [49] and Arshad et al. [31]. In this study, the dimensions of the cylinders are *R* = 0.2423 m, h=0.000648 m, and L=0.6255 m. The material properties are E=68.95 GPa and ρ=2714.5 kg/m^3^. The numerical results, which are summarized in Table 1, are close to those available in the literature.

Here, we are interested in FGM circular cylindrical shell. Thus, convergence study is performed for FGM cylinders composed of aluminum (Al) and zirconia (ZrO_2_) with CF boundary conditions. The material volume fractions are graded over the cylinder length according to a power Law (Equation (1)). The materials properties are listed in Table 2. In this study, the dimensions of the cylinders are *R* = 1 m, h/R=0.05, and L/R=10. Table 3 shows the convergence study of the fundamental frequencies with different power-law exponents (γ) and number of elements. The fundamental frequency can be considered as convergent at 36 × 40 elements (i.e., 36 elements in the circumferential direction and 40 elements in the longitudinal direction). In the following investigations, the 36 × 40 element mesh is used.

### 3.2. Parametric Study

**Figure 2:**
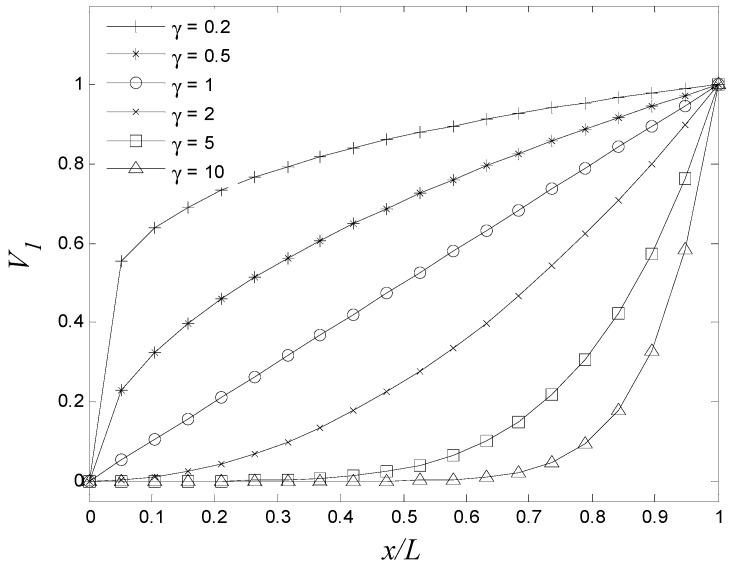
Variations of volume fractions based on simple power law (Equation (1)).

In this study, the possibility of shifting the fundamental frequency of thin cylinders using FGM graded in the axial direction is investigated. The effect of the power-law exponent (γ) on the fundamental frequency of CF thin cylinder with different dimensions is presented. The FGM compositions are aluminum (AL) and zirconia (ZrO_2_). The properties of aluminum and zirconia are listed in Table 2. The material constituents vary in axial direction as per of the power law (Equation (1)), where $V_{1}(x)$ represents the volume fraction of aluminum. The FGM cylinders have zirconia at x=0 and aluminum at x=L, as shown in Figure 2.

Table 4 shows the effect of varying the power law exponent (γ) on the fundamental frequency for different length-to-radius (L/R) ratios (assuming h/R=0.05). Table 5 shows the effect of varying the power law exponent (γ) on the fundamental frequency for different thickness-to-radius (h/R) ratios (assuming L/R=10). Similar to isotropic cylindrical shells, the fundamental frequencies of long cylinders (i.e., high L/R ratios) are smaller than those of short cylinders (i.e., increasing L/R ratio decreases the bending stiffness-to-mass ratio, which decreases the fundamental frequency). The fundamental frequency also increases by increasing the thickness (i.e., increasing h/R ratio increases the bending stiffness-to-mass ratio, which increases the fundamental frequency). Table 4 and Table 5 also show that increasing the exponent (γ) only increases the cylinder’s fundamental frequency to a certain value. Any further increase of the exponent (γ) does not increase the cylinder’s fundamental frequency, as an increase in the cylinder mass leads to the reduction in the cylinder’s fundamental frequency. This trend differs from that of FGM cylinders when material constituents vary in thickness (z). In the case of material variations in thickness, the natural frequencies of the FGM thin cylindrical shells are limited between the natural frequencies of the first- and second-base materials [31,37]. The natural frequencies depend on material distributions through the axial direction of FGM circular cylindrical shells.

### 3.3. Optimization Examples

**Figure 3:**
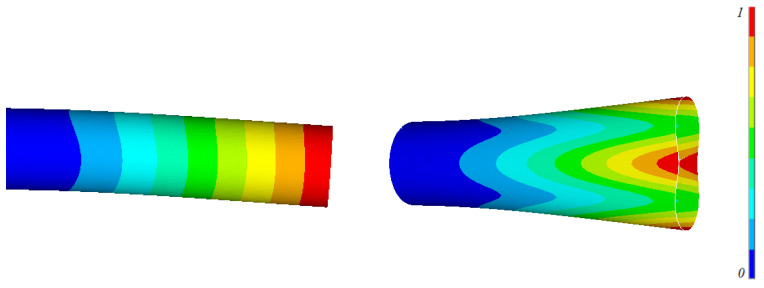
The first two mode shapes of thin clamped-free cylindrical shell.

The efficiency of using FGMs for optimizing the natural frequencies of circular cylindrical shells is demonstrated in two design examples. The first one involves maximizing the fundamental frequency of a circular cylindrical shill. In the second optimization example, the natural frequencies are shifted out of a frequency band. Practically, these design examples can be applied for cases in which we cannot control the excitation frequency (or frequencies) that happen to coincide with the cylindrical shell natural frequencies. In the optimization problems, we look for the optimal distribution of the materials’ constituents through the length of the cylindrical shells that optimize the objective function. In both examples, the cylindrical shell under consideration is a CF circular cylindrical shell (R=1 m, L/R=10, and h/R=0.05). It is composed of aluminum as the first material and zirconia as the second material. The first two natural frequencies of aluminum and zirconia cylinders are $f_{1,Al}=$ 18.84 Hz, $f_{2,Al}$ = 33.75 Hz, $f_{1,ZrO_{2}}$ = 21.54 Hz, and $f_{2,ZrO_{2}}$ = 38.59 Hz. The mode shapes are shown in Figure 3. The optimization process couples FEM and a GA to find the optimal design parameters that provide the optimal material distribution through the length of the cylinders (i.e., $V_{1}(x)$ and $V_{2}(x)$). For more information about the GA used, see Alshabatat et al. [27].

#### 3.3.1. Maximizing the Fundamental Frequency of FGM Cylindrical Shell

In most structures, the excessive vibrations occur when excitation frequencies are near the fundamental frequency. One method to solve this problem is maximizing the fundamental frequency of a structure. In this optimization problem, we look for the optimal distribution of the materials’ constituents that maximizes the fundamental frequency of a CF cylinder. The optimization of material distribution is attained by finding the optimal volume fractions at the control points. The constraint optimization problem is defined as follows:

(21) $$\begin{array}{ll} \mathrm{Maximize} & f_{1}\\ \mathrm{Design}\;\mathrm{variables} & \left\{V_{1,1},V_{1,2},\ldots ,V_{1,N}\right\}\\ \mathrm{Subject}\;\mathrm{to} & 0\leq V_{1}(x)\leq 1 \end{array}$$

**Figure 4:**
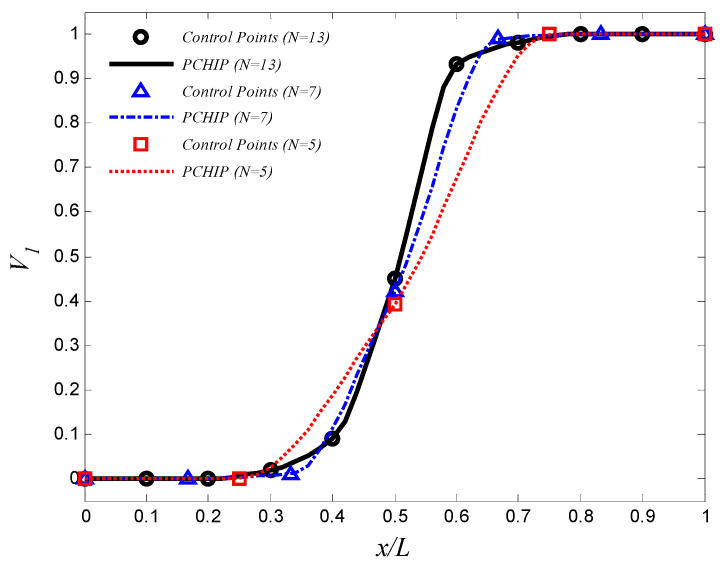
The optimal aluminum volume fraction profile which maximizes the fundamental frequency (control points approach).

**Figure 5:**
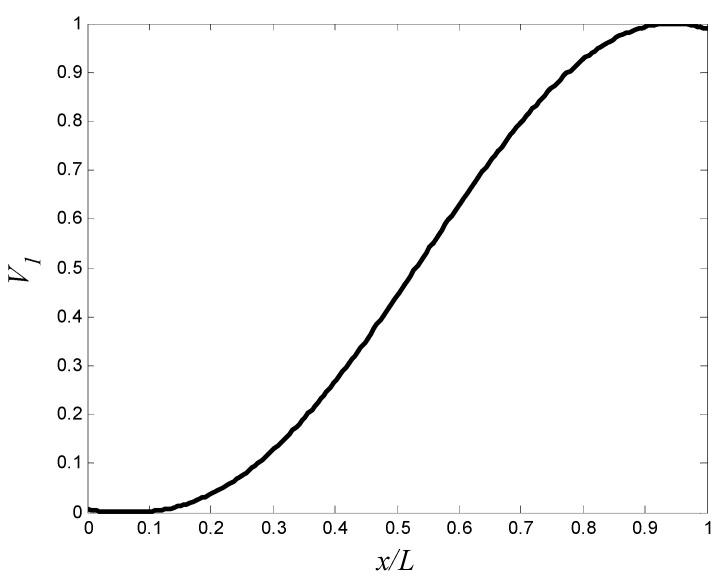
The optimal aluminum volume fraction profile which maximizes the fundamental frequency (trigonometric law, Equation (2)).

In this problem, we use different number of control points N=5,7, and 11. The volume fractions of the first material, $V_{1}(x)$, are estimated by the piecewise cubic hermit interpolating polynomial (PCHIP) [41,42]. A GA is employed to obtain the optimal design parameters $\left\{V_{1,1},V_{1,2},\ldots ,V_{1,11}\right\}$. In each generation of optimization process, the number of the population is 150. The optimization process stops when the change in the frequency is less than 10^−2^. The optimal volume fractions at the control points are summarized in Table 6, and the corresponding aluminum volume fraction distribution is shown in Figure 4. The maximum fundamental frequency is resulted by using 11 control points. The maximum fundamental frequency of the axially FGM cylinder is 29.39 Hz, which is more than the fundamental frequencies of aluminum and zirconia cylinders by 56.0% and 36.4%, respectively. To reduce the computational efforts that resulted from the high number of design variables and the interpolation method, a trigonometric law (Equation (2) is proposed to describe the volume fraction distribution in the optimization process. In this law, there are four design variables only (i.e., α,η,ϕ, and γ). The design variables must be carefully selected to ensure that $0\leq V_{1}(x)\leq 1$. The constraints are assumed as 0≤α≤0.5, −π≤η,ϕ≤π, and 0≤γ≤10. In this case, the number of the population in each generation is assumed to be 40. The optimal design parameters in Equation (2) are listed in Table 6, and the corresponding aluminum volume fraction distributions are shown in Figure 5. The maximum fundamental frequency is 28.60 Hz, which is greater than the fundamental frequency of aluminum and zirconia cylinders by 51.8% and 32.8%, respectively. Figure 4 and Figure 5 show that assigning the stiffer zirconia near the support (i.e., high bending moment region) and the lighter aluminum near the area of high modal displacement increases the fundamental frequency.

#### 3.3.2. Maximizing the Gap between Two Adjoining Natural Frequencies in FGM Cylindrical Shell

A common method to decrease vibration is by maximizing the gap between two adjoining natural frequencies to avoid the coincidence of these natural frequencies with the excitation frequencies that lie between them [50]. In this optimization problem, we look for the volume fractions of the materials’ constituents to maximize the distance between the first and second natural frequencies of the CF cylinder. The cylinder under discussion is similar in size and base materials to one in the previous example. We assume that the cylinder is exposed to a force with excitation frequencies in the range of 15–35 Hz. As mentioned previously, the first and second natural frequencies of the aluminum cylinder are $f_{1,Al}=$ 18.84 Hz and $f_{2,Al}$ = 33.75 Hz, and the first natural frequency of the zirconia cylinder is $f_{1,ZrO_{2}}$ = 21.54 Hz. These natural frequencies are located in the frequency band of the force. Thus, our target is to shift the natural frequencies out the range of 15–35 Hz. This constraint optimization problem can be stated as follows:

(22) $$\begin{array}{ll} \mathrm{Maximize} & f_{2}-f_{1}\\ \mathrm{Design}\;\mathrm{variables} & \left\{V_{1,1},V_{1,2},\ldots ,V_{1,N}\right\}\\ \mathrm{Subject}\;\mathrm{to} & 0\leq V_{1}(x)\leq 1\\ & f_{1}<15\mathrm{Hz}\\ & f_{2}>35\mathrm{Hz} \end{array}$$

**Figure 6:**
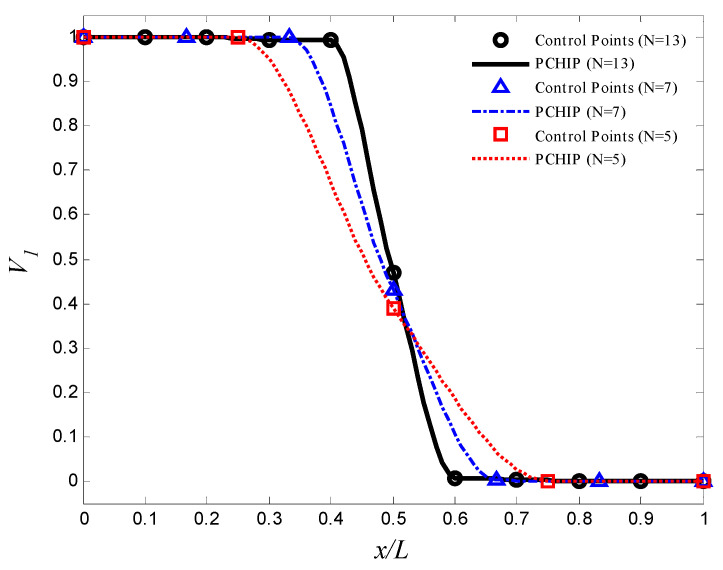
The optimal aluminum volume fraction profile which maximizes the gap between $f_{1}$ and $f_{2}$ (control points approach).

**Figure 7:**
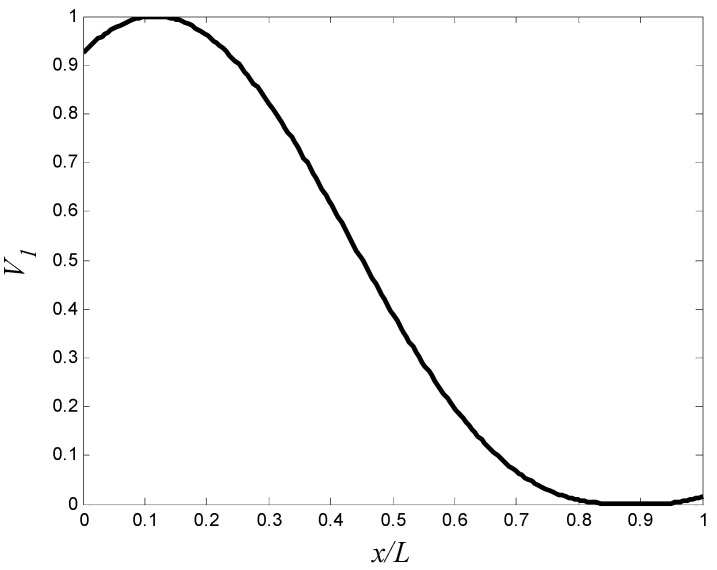
The optimal aluminum volume fraction profile which maximizes the gap between $f_{1}$ and $f_{2}$ (trigonometric law, Equation (2)).

The GA parameters are similar to those in the previous example. The optimal volume fractions at the control points are summarized in Table 7, and the corresponding aluminum volume fraction distributions that maximizes the gap between the natural frequencies are shown in Figure 6. The optimal frequencies are summarized in Table 7. The maximum gab between the first and second natural frequencies is resulted by using 11 control points. The gap between the first and second natural frequencies of the optimal FGM cylinder is 24.64 Hz, which is greater than the gap between the first and second natural frequencies of aluminum and zirconia cylinders by 65.3% and 44.5%, respectively. By using the trigonometric law (Equation (2)), with similar constraints, number of the population, and stopping criterion to the previous example, the optimal gap between the first and second natural frequencies of the FGM cylinder is 23.84 Hz, which is more than the gap between the first and second natural frequencies of aluminum and zirconia cylinders by 59.9% and 39.8%, respectively. the optimal design variables are listed in Table 7, and the corresponding aluminum volume fraction distribution is shown in Figure 7.

Figure 6 and Figure 7 show that assigning the low stiffness aluminum near the support (i.e., high bending moment region in the first mode) decreases the first natural frequency. In addition, assigning the high stiffness zirconia at the area of high modal displacement in the second mode outweighs the disadvantage of increasing masshus. The second natural frequencies of the optimized cylinders are close to natural frequency of zirconia cylinder.

Using the control points method in material distribution optimization is efficient for finding the optimal natural frequencies, but it has a large number of design variables, making it computationally expensive. Moreover, the high number of control points may cause a very high gradient of volume fraction. This high gradient may lead to a sudden change in mechanical properties, which causes cracks and delamination. On the contrary, the trigonometric law can achieve good optimization results with acceptable computational efforts.

## 4. Conclusions

In this article, a technique to optimize the natural frequencies of circular cylindrical shells using FGM was presented. The constituents of the FGM were graded in the axial direction. Material volume fraction distribution was described by simple power law, four-parameter trigonometric law, and piecewise cubic interpolation of volume fractions at control points. The effective material properties were estimated by applying Voigt model. FSDT and FEM were employed for free vibrations analysis. To validate the FE program, numerical results were compared with the results of other authors. Two optimization problems were presented. The first problem focused on maximizing the fundamental frequency of a clamped-free FGM circular cylindrical shell by optimizing the distribution of aluminum and zirconia through the axial direction. The maximum fundamental frequency, based on the control points method, was 56.0% and 36.4% more than the fundamental frequencies of aluminum and zirconia cylinders, respectively. In the second example, the gap between the first and second natural frequencies of a FGM circular cylindrical shell was maximized. Based on the control points method, the maximum gap between these frequencies was 65.3% and 44.5% more than those of aluminum and zirconia cylinders, respectively. It was found that using light material near the area of high modal displacement and using stiff material near the area of high bending moment increased the corresponding natural frequency. The control points method is efficient in optimizing the natural frequencies. Increasing the number of control points gave better results. However, the high number of control points may cause a very high gradient of volume fraction which may cause cracks and delamination in real life cases. In addition, the high number of control points makes the design method computationally expensive. Thus, a good alternative is to use the suggested trigonometric law. (Equation (2)) to reduce the drawbacks of control points method. This law provides a flexible description of material volume fraction profile with small number of design variables, which gives engineers a powerful tool for design optimization with accepted computational efforts.

The presented examples show the capabilities of axially FGM in optimizing the natural frequencies of cylindrical shells. A further study of the proposed method is recommended to take in consideration other boundary conditions. Future extension of this work may include minimizing the vibration and sound radiation from such structures.

## Figures and Tables

**Table 1:** Comparison of the lowest natural frequencies of clamped-free isotropic cylindrical shells.

		Frequency (Hz)				
	Elements	1	2	3	4	5
Present	20 × 10	94.08	108.09	108.6	138.6	167.3
	24 × 10	93.26	106.25	108.49	135.29	167.78
	36 × 20	92.58	105.28	108.15	133.95	168.28
Ref. [49]		92.55	105.05	108.47	133.60	169.12
Ref. [31]		95.38	106.33	114.39	134.23	171.84

**Table 2:** Material properties of aluminum and zirconia.

Property	Al	ZrO_2_
*E* (GPa)	20	205
*ρ* (kg/m^3^)	2700	6050
*v*	0.3	0.31

**Table 3:** Convergence of the fundamental frequency (Hz) of clamped-free FGM cylinders with number of finite elements.

	γ		
Elements	0.5	1	10
12 × 5	23.67	24.88	21.84
24 × 5	24.26	25.47	22.38
36 × 5	24.57	25.80	22.67
36 × 10	25.17	26.40	23.19
36 × 20	25.32	26.53	23.35
36 × 40	25.34	26.54	23.37

**Table 4:** Variations of the fundamental frequencies (Hz) with the power-law exponent and length to radius ratio (L/R ).

		γ						
L/R	Al	0.5	1	2	5	10	100	ZrO_2_
0.2	1347.0	1689.1	1758.1	1754.9	1678.2	1622.3	1549.0	1539.9
0.5	469.0	559.8	581.0	586.6	572.9	559.1	538.77	536.21
1	231.0	282.6	293.2	296.1	286.1	277.4	265.54	264.09
2	119.3	136.1	140.6	142.3	140.7	139.1	136.7	136.4
5	41.45	47.71	49.31	49.76	49.05	48.40	47.50	47.39
10	18.84	25.35	26.54	26.33	24.63	23.38	21.75	21.54
15	8.68	11.75	12.31	12.20	11.38	10.79	10.02	9.92
20	4.94	6.71	7.03	6.97	6.50	6.16	5.71	5.65

**Table 5:** Variations of the fundamental frequencies (Hz) with the power-law exponent and thickness to radius ratio (h/R ).

		γ						
h/R	Al	0.5	1	2	5	10	100	ZrO_2_
0.001	3.52	4.38	4.50	4.57	4.43	4.27	4.05	4.02
0.005	7.09	9.17	9.57	9.52	9.01	8.64	8.16	8.10
0.01	9.13	10.92	11.33	11.37	11.04	10.80	10.48	10.44
0.02	14.68	16.14	16.59	16.83	16.87	16.85	16.80	16.79
0.03	18.82	22.26	22.79	23.23	23.57	23.36	21.73	21.52
0.04	18.83	25.34	26.53	26.32	24.62	23.37	21.74	21.53
0.05	18.84	25.34	26.54	26.33	24.63	23.38	21.75	21.54

**Table 6:** The optimal frequencies and design variables of the first design problem.

Material Type	$f_{1}(\mathrm{Hz})$	Optimal Design Variables
Aluminum	18.84	-
Zirconia	21.54	-
FGM (5 Control Points)	28.93	{0,0,0.39,1,1}
FGM (7 Control Points)	29.25	{0,0,0.01,0.42,0.99,1,1}
FGM (11 Control Points)	29.39	{0,0,0,0.019,0.090,0.450,0.931,0.982,1,1,1}
FGM (Equation (2))	28.60	α=0.5 , η=−0.397 , ϕ=−2.813 , γ=1.351

**Table 7:** The optimal frequencies and design variables of the second design problem.

Material Type	$f_{1}(\mathrm{Hz})$	$f_{2}(\mathrm{Hz})$	$f_{2}-f_{1}(\mathrm{Hz})$	Optimal Design Variables
Aluminum	18.84	33.75	14.91	-
Zirconia	21.54	38.59	17.05	-
FGM (5 Control Points)	13.87	37.94	24.07	{1,1,0.39,0,0}
FGM (7 Control Points)	13.57	37.95	24.38	{1,1,1,0.43,0.01,0,0}
FGM (11 Control Points)	13.32	37.96	24.64	{1,1,1,0.995,0.994,0.469,0.007,0.004,0,0,0}
FGM (Equation (2))	14.13	37.97	23.84	α=0.5 , η=−0.406 , ϕ=0.473 , γ=1.381

## Data Availability

The data presented in this study are available on request from the author.
